# NDRG1 regulates osteosarcoma cells via mediating the mitochondrial function and CSCs differentiation

**DOI:** 10.1186/s13018-021-02503-5

**Published:** 2021-06-07

**Authors:** Tong Zhao, Ying Meng, Yongping Wang, Wenji Wang

**Affiliations:** 1 The First Hospital of Lanzhou University, No. 1 Dongggang West Road, Chengguan District, Lanzhou, Gansu China; 2 Lanzhou University Second Hospital, Lanzhou, Gansu China

**Keywords:** Cancer stem cell, N-myc downstream regulated 1, Osteosarcoma, Mitochondria-mediated apoptosis

## Abstract

**Background:**

Cancer stem cells (CSCs) are mainly contributed to malignancy metastatic potential and resistant therapy of osteosarcoma (OS). The mitochondria-related apoptosis was generally accepted as the target of tumor therapy. However, the effect of N-myc downstream-regulated gene 1 (NDRG1) on CSCs and mitochondrial health in OS is still unknown.

**Methods:**

In OS cells, MG63 and U2OS, the siRNA of NDRG1 were conducted. Transwell, western blot, RT-qPCR, and mitochondria isolation were used to identify the effect of NDRG on OS cells and mitochondria. Moreover, the differentiation-related factors of CSCs were determined.

**Results:**

After downregulation of NDRG1, the cell viability, invasion ability decreased whereas cell apoptosis increased. The expressions profiles of fibronectin, vimentin, vascular endothelial growth factor (VEGF), matrix metalloproteinase (MMP) 2, MMP9, and MMP13 were downregulated, but E-cadherin expression level was upregulated by NDRG1 siRNA. At the same time, cytochrome (Cyt) C levels were increased in cytosol with the decreasing in mitochondria after siRNA treatment. The mitochondrial membrane potential (MMPs) was declined, and the function of mitochondria was impeded. The expressions of uncoupling proteins (UCP) 2, voltage dependent anion channel (VDAC), peroxisome proliferator-activated receptor gamma coactivator (PGC)-1α, and cyclooxygenase (COX) 2 were downregulated by NDRG1 silencing. Moreover, NDRG performed its function primarily through the Wnt pathway and could regulate the differentiation of osteosarcoma stem cells.

**Conclusion:**

Silencing of NDRG1 could damage the function of mitochondria, promote the CSCs differentiation, alleviating OS progression.

## Introduction

Osteosarcoma (OS) is well known as a primary, high malignancy, and metastatic potential bone tumor [1, 2]. Although the use of neoadjuvant chemotherapy has improved the 5-year survival rate of OS patients, the prognosis remains poor. Therefore, identifying new early diagnostic biomarkers and therapeutic targets are essential research goals for OS.

The metastasis inhibitor N-myc downstream-regulated gene 1 (NDRG1) is a stress response protein that is involved in the inhibition of multiple oncogenic signaling pathways. NDRG1 expression was regulated by multiple factors in both healthy and cancerous cells [3], and abnormal expression affected cell proliferation, differentiation [4, 5], migration, invasion, and stress responses [6]. The overexpression of NDRG1 was induced by hypoxia in various human cancers [7], like lung cancer, liver cancer, and brain cancer.

In recently, research found that regulation of NDRG1 could inhibit HGF and IGF-1signal, reduce cell migration and enhance the drug sensitive in pancreatic cancer, and NDRG1 expression could be repressed by miR-1469-5p, regulating NF-ƙB pathway activity [8, 9]. In prostate cancer, NDRG1 is phosphorylated by PIM1 to enhanced cell migration and invasion [10]. Cell apoptosis was induced by NDRG1 downregulation in HCC, and mitochondrial damage were induced by the upregulation of BAX and downregulation of Bcl-2 and Bcl-x [11]. Importantly, research found that NDRG1 promotes the stem-like properties of lung cancer cells through Skp2-mediated ubiquitination preventing the degradation of c-Myc [12]. NDRG1 overexpression promotes the progression of esophageal squamous cell carcinoma through regulating Wnt signaling pathway [13]. NDRG1 also regulated the molecular motor, decreased the migration ability, playing a role of anti-transfer in tumor cells [14]. The role of NDRG1 on OS cells was rarely revealed so far. Researches showed that NDRG1 expression abrogation sensitized OS cells to chemotherapy, increasing cells apoptosis [15].

According to previous researches, we hypothesized that the mitochondrial function and CSC differentiation could be regulated by changing the levels of NDRG1, alleviating the progression of OS. The aim of this study is to investigate the role of NDRG1 on mitochondria and CSCs of OS.

## Methods

### Cell culture

The human osteoblast cell line hFOB, human OS cell lines U2OS, and MG63 were purchased from the Shanghai Cell Bank of the Chinese Academy of Sciences. The cells were cultured at 37 °C and 5% CO_2_, the medium of the cells respectively were DMEM/F12 (Hyclone) and RPMI 1640 (Hyclone), respectively and containing 10%FBS (Gibco) and penicillin and streptomycin (100 U/mL, Gibco). Cells were passaged by digestion with 0.25% trypsin.

### RNA interference

The *NDRG1* (NCBI Accession No: NG_007943.1) sequence was obtained from GenBank, and used to design the *NDRG1* siRNA sequence. Cells (4 × 10^6^ cells/mL) were washed with 1 mL of serum-free medium, passed on to 6-well cell culture plates. After incubating for 24 h, cells were transfected with NDRG1 siRNA and negative control (NC) and incubated with transfection reagent according to manufacturers’ instructions. The transfection mixture was added to the wells, and the cell culture medium was changed after 24 h. After 72 h, cells were treated with 2.5 mg/mL puromycin for 96 h to select for stable transfection. Downregulation of NDRG1 expression was confirmed.

### Viability assays

Single cell suspensions were prepared, and inoculated into 96-well plates. The corresponding treatments were added to each group of cells before 24 h incubation. Cells were then incubated with MTT (Beyotime), then culture medium was discarded, and the OD at 570 nm was measured.

### Invasion assays

Transwell chambers were placed in 24-well cell culture plates. Plates were incubated at 37 °C and 5% CO_2_ for 24 h, containing single-cell suspension in upper chamber and medium supplemented with serum in lower chamber. Cells were washed, then fixed. A cotton swab was used to gently scrape off cells that did not migrate. Cells were stained with 0.1% crystal violet. The migration status of the cells was observed with a microscope.

### Mitochondrial isolation

The isolation of mitochondria was performed according to the instructions of kits (Sigma). Namely, the cells were subjected to wash with phosphate buffer solution. Cells were resuspended with lysis buffer. After that, 1× extraction buffer was added before centrifuge. Then, the pellet was obtained.

### JC-1 assay

The cells were incubated with JC-1 solution (Beyotime) at 37 °C, 30min. A microreader was used to obtain the fluorescence of MMPs at 540 and 490 nm.

### Flow cytometry

CD133+ positive cells were enriched by flow separation using magnetic activated cell sorting. Simply, sorting buffer was used to prepare cell suspension with density of 5 × 10^6^ cells/mL. Subsequently, microbeads combined with mouse anti-Human CD133 antibody were added and incubated for 30 min. Cells were washed and resuspended to separation.

### Sphere formation assay

CD133+ cells were cultured in Dulbecco’s Modified Eagle Medium/F12 medium plus with 20 ng/ml EGF, 20 ng/ml bFGF, 0.4% BSA, and 2% B27 for 500 cells/well. Tumor-sphere formation was used to identify CD133+ isolation.

### Western blot

Proteins were extracted using radioimmunoprecipitation assay lysis buffer. After separation by sodium dodecyl sulfate polyacrylamide gel electrophoresis, the proteins were transferred to PVDF membrane, and blocked in 5% skim milk in PBS containing 0.5% Tween 20 for 1 h, then the membrane was incubated with primary antibodies and secondary antibodies, and visualized by electrochemiluminescence. Semiquantitative grayscale data analysis was performed.

### qRT-PCR

RNeasy Plus Universal Kits (QIAGEN) were used to extract total RNA of the cells. RNA purity and concentration were determined using a NanoDrop 2000 spectrophotometer. Reverse transcription was carried out using cDNA synthesis kit (QIAGEN). The quality of the data obtained was confirmed based on the amplification and melting curves. Relative mRNA expression was calculated using the formula F = 2^−ΔΔCt^. Primer sequences are shown in Table 1.

**Table 1:** Primers sequences

Gene	Forward	Reverse
ß-catenin	TGGTGCCCAGGGAGAACCCC	CCCACCCCTCGAGCCCTCTC
NDRG1	GGATCAGTTGGCTGAAAT	ATCTTGAGTAGGGTGGTCTT
Bax	GGAGCTGCAGAGGATGATTG	CCAGTTGAAGTTGCCGTCAC
Bcl-2	CTGAGGAGCTTTGTTTCAACCA	TCAAGAAACAAGGTCAAAGGGA
Fibronectin	GGAGCAAATGGCACCGAGATA	GAGCTGCACATGTCTTGGGAAC
Vimentin	TGCCGTTGAAGCTGCTAA CTA	CCAGAGGGAGTGAATCCAGAT TA
VEGF	TTGCCTTGCTGCTCTACCTCCA	GATGGCAGTAGCTGCGCTGATA
E-cadherin	AATGCCGCCATCGCTTAC	AGTTCGAGGTTCTGGTAGGG
TCF 4	TGGCCCTGAGAGGCAGCCAT	GGTCCTCATCGTCATTATTGCTAGAT
LEF 1	CCAGCTATTGTAACACCTCA	TTCAGATGTAGGCAGCTGTC
LRP 5	CAGCCTGACGCACCCCTTCG	CACCTCCTCGGCTCCTGCCT
LRP 6	GCTGGAATGGATGGTTCAAGTCG	CAGAATGGATTTCACGCAGACCC
Wnt 3a	GTTGGGCCACAGTATTCCTC	ATCCCACCAAACTCGATGTC
Runx2	AAGCTTGATGACTCTAAACC	TCTGTAATCTGACTCTGTCC
Osterix	TGAGGAGGAAGTTCACTATG	CATTAGTGCTTGTTAAAGGGG
Dlx5	GCATTACAGAGAAGGTTTCAG	TTTTCACCTGTGTTTGTGTGTC
Coll I	GCTATGATGAGAAATCAACCG	TCATCTCCATTCTTTCCAGG
ß-actin	TGGCATTGCCGACAGGATGCAGCAA	CTCCTCATACTCCTGCTTGCTGAT

### Statistical analysis

All data in this study were analyzed using the SPSS 20.0 software. The t test and one-way analysis of variance were used to analyze statistical differences. *P* values < 0.05 were considered to indicate statistically significant results. n.s. means p > 0.05, * mean p < 0.05,** means p < 0.01.

## Results

### Silence of NDRG1 inhibited the proliferation and invasion of OS cells

**Fig. 1:**
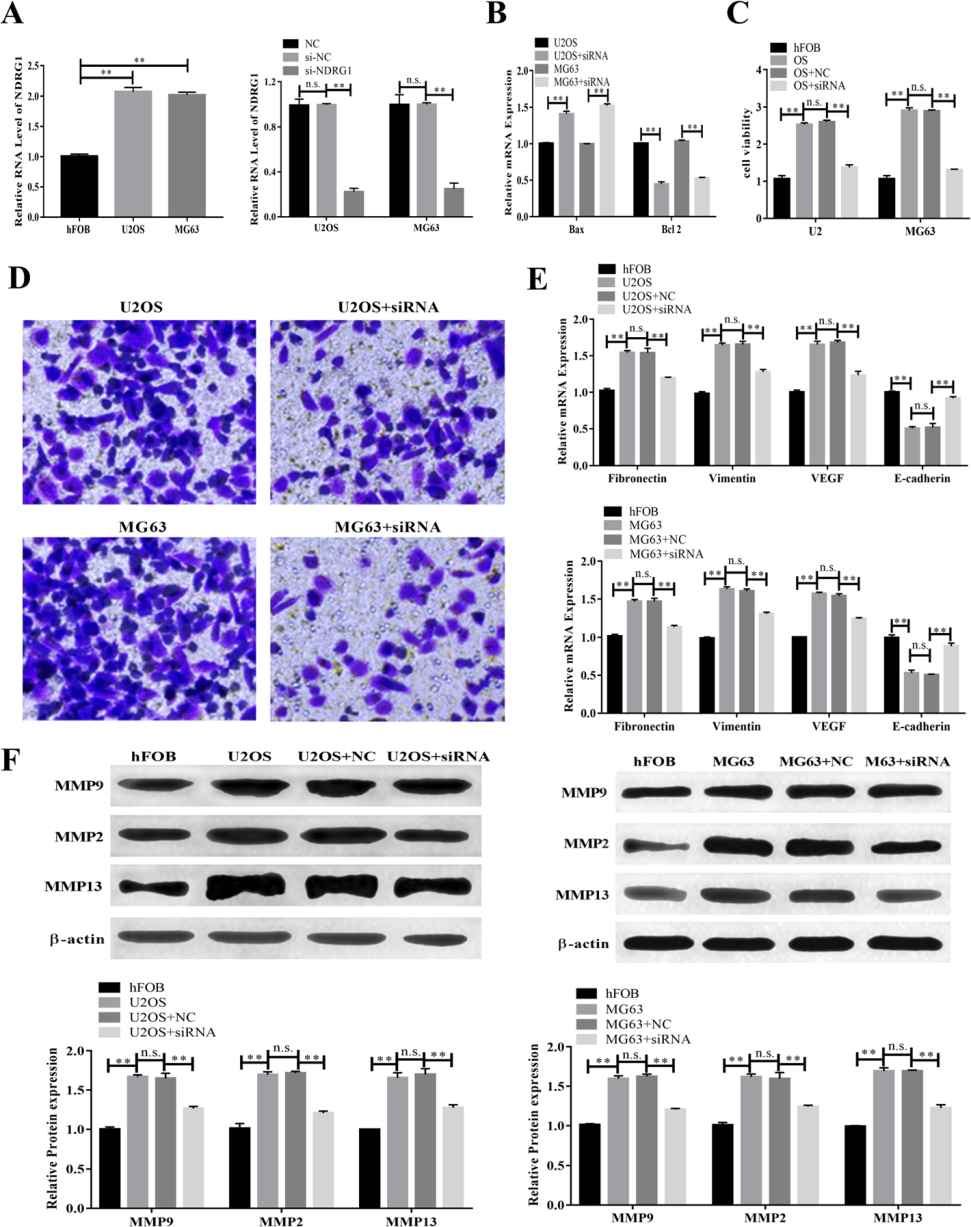
Downregulation of NDRG1 expression promoted osteosarcoma cells migration, invasion. (**A**) The expression of NDRG after siRNA in hFOB, U2OS, and MG63. (**B**) Effect of NDRG1 on cell apoptosis. (**C**) NDRG expression enhanced the OS cells viability. (**D**) NDRG positively correlated with OS cells invasion. (**E**) The mRNA levels of VEGF, E-cadherin, fibronectin, and vimentin were decreased by NDRG siRNA. (**F**) Protein levels of MMP2, MMP9, and MMP13 were downregulated by siRNA

In this study, the expression levels of NDRG1 in U2OS and MG63 were higher than hFOB, while the siRNA decreased the level of NDRG1 (p < 0.01). After transfection, the levels of NDRG1 in si-NC and NC were tripled comparing with si-NDRG1 (Fig. 1A). U2OS and MG63 cells apoptosis increased, cell viability and invasion ability decreased by siRNA (Fig. 1B, C, D). After siRNA depletion of NDRG1 in OS cells, the expression levels of VEGF, fibronectin, and vimentin were significantly decreased, whereas E-cadherin expression was significantly increased (all *p* < 0.01; Fig. 1E). At the same time, the expression of MMP2, MMP9, and MMP13 was decreased when NDRG1 silencing (Fig. 1F). The results showed that siRNA interference inhibited OS cells viability, invasion, and migration progression.

### Downregulation of NDRG1 promoted mitochondria-mediated apoptosis

**Fig. 2:**
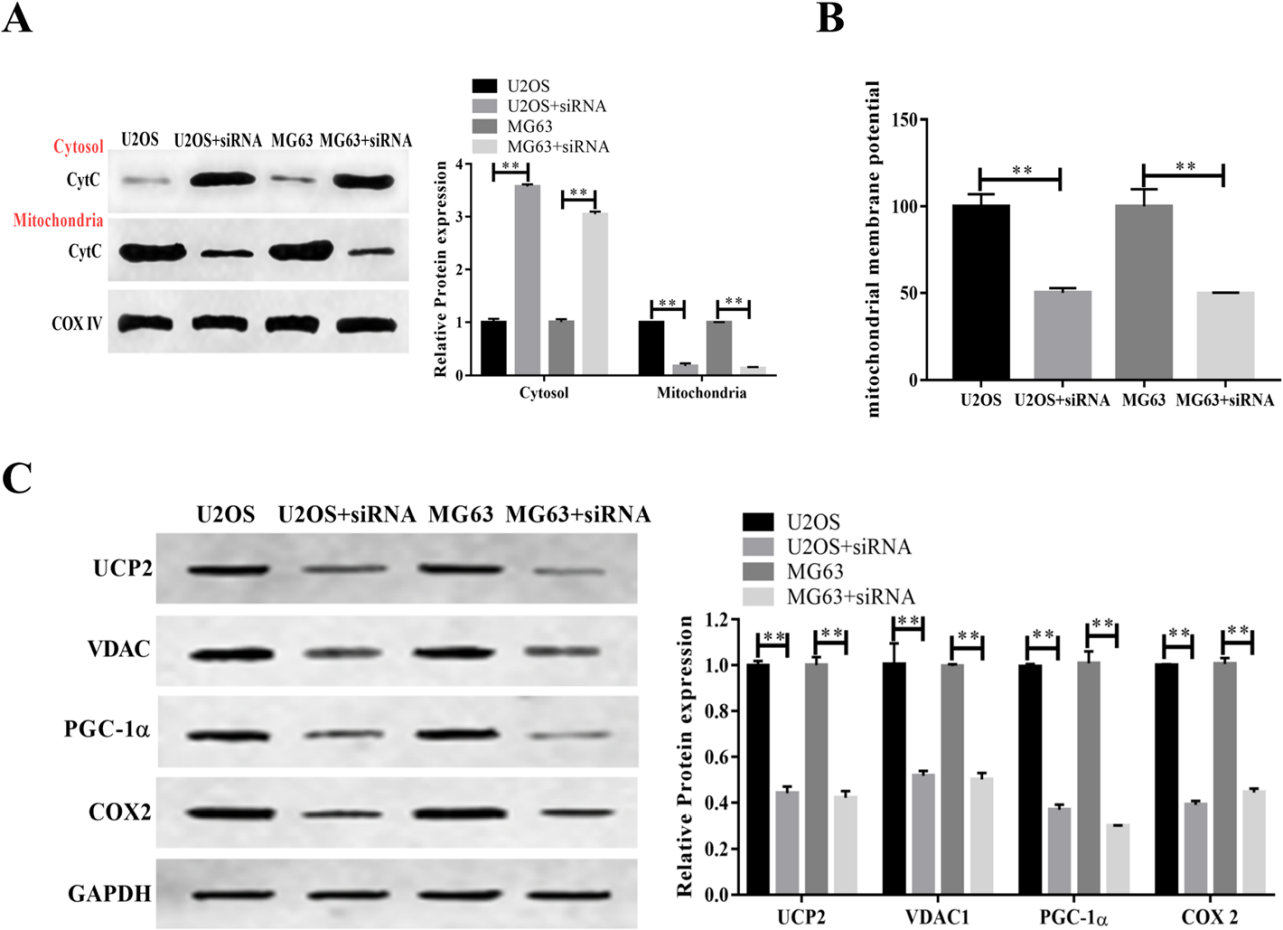
Inhibition of NDRG activated the mitochondria-mediated apoptosis. (**A**) Changes of CytC in cytosol and mitochondria. (**B**) Mitochondrial membrane potential was declined when NDRG expression was inhibited. (**C**) UCP2, VDAC, PGC-1α, and COX2 were downregulated by siRNA

The apoptosis of mitochondria-mediated was the leakage of CytC. Compared with control, U2OS and MG63, the levels of CytC were significantly increased in cytosol of cells subject to NDRG siRNA (Fig. 2A). Structure and function of mitochondria were affected by NDRG1 expression. The mitochondrial membrane potentials were declined, and UCP2, VDAC, PGC-1α, and COX2 were downregulated (Fig. 2B, C).

### NDRG1 regulated CSCs differentiation via Wnt signal

**Fig. 3:**
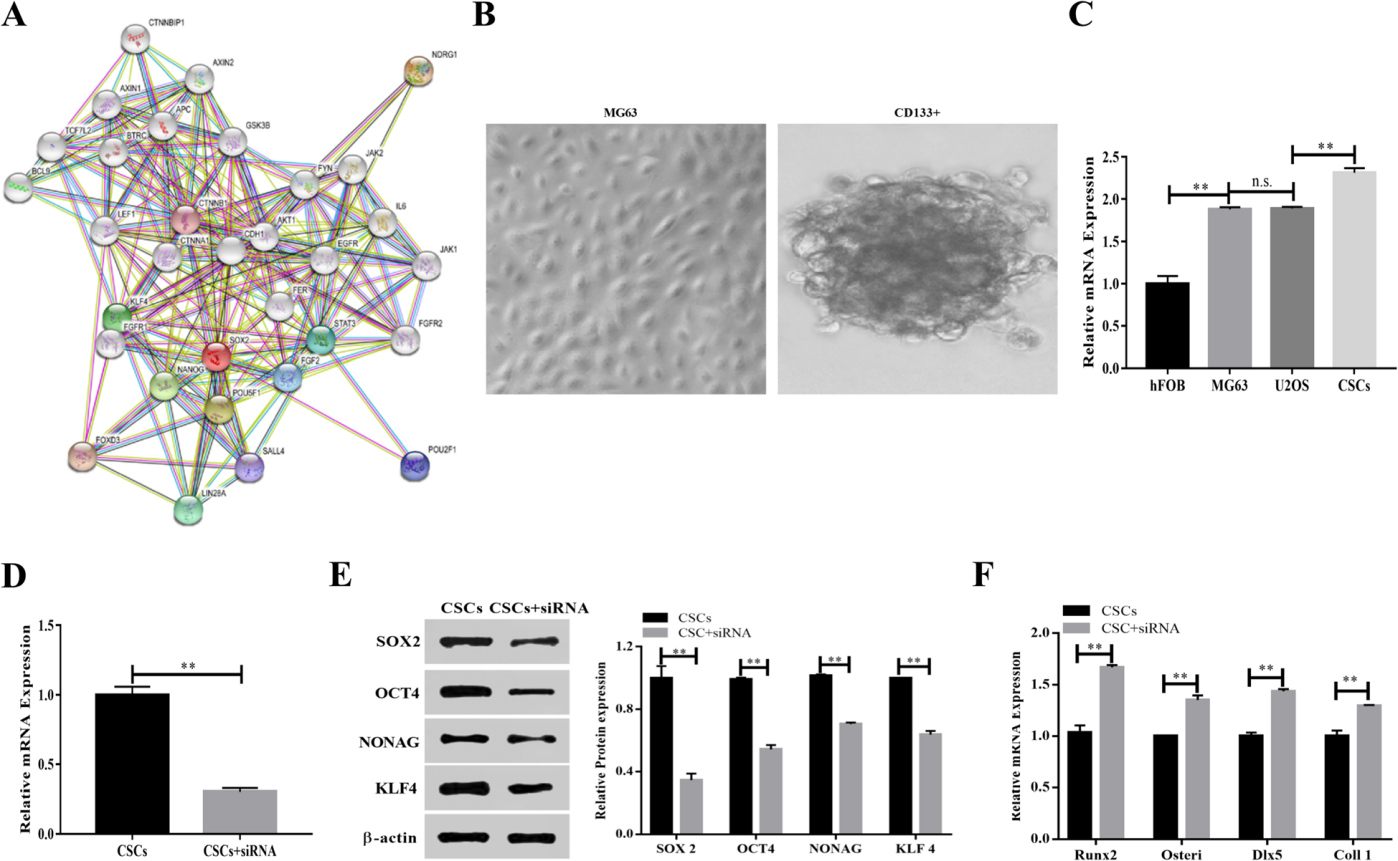
NDRG1 expression maintained the CSCs stemness. (**A**) STRING was used to predict interaction between NDRG and SOX2. (**B**) Tumor-formation assay for CD133+ positive cells. (**C**) The expression of NDRG1 in different cell lines. (D) NDRG1 siRNA downregulated NDRG1 in CSCs. (**E**) Silencing of NDRG1 reduced the pluripotency of CSCs. (**F**) Downregulation of NDRG1 promoted CSCs differentiation

**Fig. 4:**
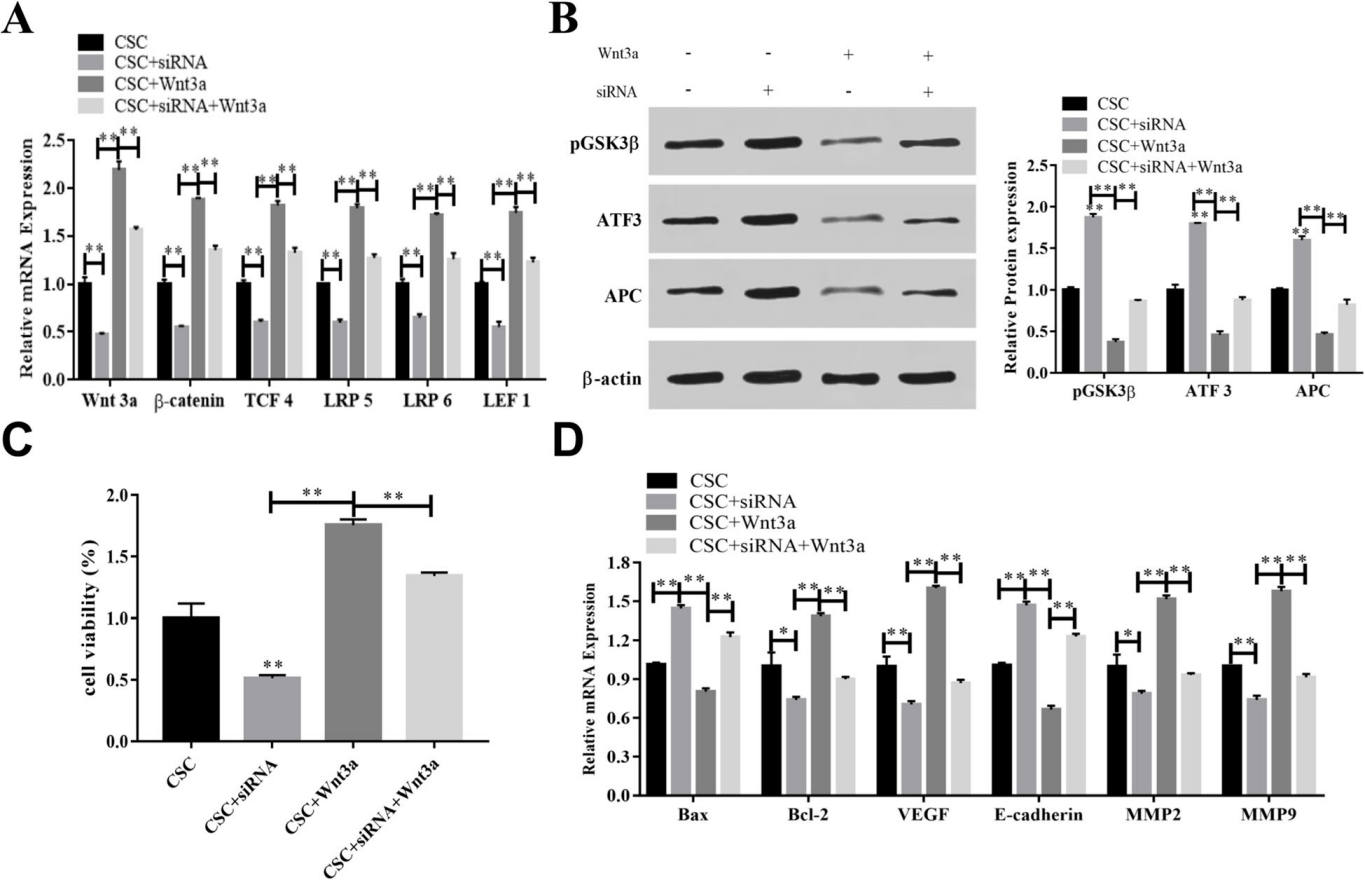
NDRG played a role on CSCs via Wnt signal. (**A**) The mRNA levels of Wnt3a, ß-catenin, TCF4, LRP5/6, and LEF reduced by NDRG siRNA. (**B**) The content of pGSK3ß, ATF3, and APC were determined by western blot. (**C**) Effect of Wnt signal and NDRG1 on cell viability. (**D**) The activation of Wnt signal promoted cell proliferation, migration

The database of STRING was applied to predict the interaction between NDRG1, pluripotency, and Wnt signal (Fig. 3A). To demonstrate the effect of NDRG1 on OS CSCs, CD133+ positive cells were enriched by flow sorting marked CD133. Tumor-sphere formation demonstrated successful enrichment of CD133+ (Fig. 3B). In different cell lines, the expression of NDRG1 was difference. The NDRG1 levels were enriched in CSCs further, and were higher than hFOB, MG63, and U2OS cells (p < 0.01, Fig. 3C). And the siRNA of NDRG1 decreased the expression of NDRG1 (Fig. 3D). Compared with CSCs control group, the expression levels of Runx2, Osteri, Dlx5, Coll I were upregulated by NDRG1 siRNA, promoting the differentiation of CSCs, while sex determining region Y-box (SOX) 2, octamer-binding transcription factor (OCT) 4, NONAG, and kruppel-like factor (KLF) 4 expression levels were downregulated (Fig. 3E, F). The downregulation of NDRG1 in CSCs inhibited activation of Wnt signaling pathway. After NDRG1 downregulation, Wnt pathway activation was receded. The expression of wingless-type MMTV integration site family member (Wnt) 3a, ß-catenin, transcription factor (TCF) 4, LRP5/6, and lymphoid enhancer binding factor (LEF) 1 were decreased by siRNA. However, the recombination protein of Wnt3a played the opposite effect comparing with NDRG1 siRNA, the combination treatment of Wnt3a and siRNA neutralizes the role of siRNA and Wnt3a (Fig. 4A, B). The activation of Wnt signal promoted the proliferation of CSCs, the cell apoptosis was reduced, and the expression of VEGF, MMP2, and MMP9 were upregulated. The treatment of si-NDRG1 increased the cell apoptosis, E-cadherin expression was upregulated (Fig. 4C, D).

## Discussion

The cellular heterogeneity was observed in OS tumor samples and the presence of self-renewing subpopulations that did not respond to chemotherapy, which was attributed to CSCs [16]. These cells were characterized by the expression of pluripotency-related markers, such as SOX2 [17] and activation of signaling pathways that controlled stem cell self-renewal. Studies have found that all-trans retinoic acid inhibited the upregulation of CD117-Stro1 cells and CSC markers by M2-like macrophages, prevented the malignant transformation and differentiation of OS cells [18]. Most OS tumors are relatively undifferentiated. Therefore, induction of differentiation may be an interesting therapeutic strategy in that differentiated cells that may be more amenable to treatment. SOX2 is an essential factor in maintaining the undifferentiated state of OS cells and is critical for their self-renewal. And it is also an antagonist of the Wnt pathway. Studies have shown that the tankyrase inhibitor JW74, which attenuated Wnt/β-catenin activity, and induced U2OS cell differentiation, even though these cells were resistant to osteogenic differentiation under standard conditions [19].

NDRG1 played a role in OS cell differentiation and invasion [20], including inhibited tumor growth by modifying angiogenesis by reducing expression of the angiogenic gene VEGF [21]. NDRG1 expression was associated with MMP-2, -9, -10, and BCL2 apoptosis [22]. Recently, it was verified that the abnormal expression of NDRG4 was significantly associated with the biological function like ATP synthesis and mitochondrial membrane potential [23]. And the tumorigenesis potential of GBM CSCs was related to NDRG4 [24]. Moreover, the regulation of mitochondrial alternative oxidase expression could affect cell migration by changing of mitochondrial heat production [25]. The results of those researches were consistent with our primary hypothesis.

In the present study, the expression of NDRG1 was downregulated by siRNA treatment, and cell apoptosis increased. The protein expression of VEGF, E-cadherin, fibronectin, vimentin, and MMP were reduced. At the same time, mitochondria-dependent apoptosis was also induced by silencing of NDRG1, CytC was leaked in mitochondria and increased in cytosol. And the function of mitochondria was attenuated. In CSCs enriched by CD133-positive, the levels of SOX2, OCT4, NONAG, and KLF4 were bated by NDRG1 siRNA, accompanying the expression upregulation of differentiation related factors. The downregulation of NDRG1 inhibited the abnormal Wnt activation. Therefore, this research suggested that downregulation of NDRG1 inhibited the mitochondrial function and cell proliferation, migration. And the downregulation of NDRG1 promoted CSCs differentiation via regulating Wnt signal.

## Conclusion

NDRG1 downregulation hampered the mitochondrial function, and decreased the invasive and metastatic ability of OS. In addition, silencing of NDRG1 reduced abnormal Wnt activation in tumors primarily, which promoted OS CSC differentiation, thus playing a role in regulating the cell cycle.

## Data Availability

The datasets supporting the conclusions of this article are included within the article.

## Abbreviations

CSCs: Cancer stem cells
OS: Osteosarcoma
NDRG1: N-myc downstream regulated gene 1
VEGF: Vascular endothelial growth factor
MMP2: Matrix metalloproteinase 2
MMPs: Mitochondrial membrane potential
UCP2: Uncoupling proteins 2
VDAC: Voltage dependent anion channel
PGC-1α: Peroxisome proliferator-activated receptor gamma coactivator
COX2: Cyclooxygenase 2
CytC: Cytochrome C
SOX2: Sex determining region Y-box 2
OCT4: Octamer-binding transcription factor 4
KLF4: Kruppel-like factor 4
Wnt3a: Wingless-type MMTV integration site family member 3a
TCF4: Transcription factor 4
LEF1: Lymphoid enhancer binding factor 1
